# Ecomorphological Convergence Following Niche Shifts in Montane Ground Beetles (Carabidae: *Nebria*)

**DOI:** 10.1002/ece3.70986

**Published:** 2025-02-19

**Authors:** Jillian K. Schat, Elizabeth Ehlert, David H. Kavanaugh, Roman Yu Dudko, Sean D. Schoville

**Affiliations:** ^1^ Department of Entomology University of Wisconsin Madison Madison Wisconsin USA; ^2^ California Academy of Sciences, Department of Entomology San Francisco California USA; ^3^ Institute of Systematics and Ecology of Animals Siberian Branch of the Russian Academy of Sciences Novosibirsk Russia; ^4^ Tomsk State University Tomsk Russia

**Keywords:** adaptive radiation, alpine, Carabidae, convergent evolution, ground beetles, morphology

## Abstract

A critical step toward uncovering generalizable patterns of phenotype‐niche relationships is understanding how functional traits have evolved as species occupy new habitats. Ecomorphological traits impact how organisms function in their environment and are predictive of habitat use and niche. Studying ecomorphological variation in the context of strong environmental filtering can provide opportunities to understand the role of convergent evolution in forming trait‐habitat use patterns. By integrating a molecular phylogeny, habitat use, and morphometrics, this study aimed to understand the role of ancestry and convergent evolution in ecomorphological trait evolution. This study examined these processes using an assemblage of 79 species in the ground beetle genus *Nebria* (Carabidae: Nebriini). Species in this genus are habitat specialists who occupy montane and alpine streambeds, talus, and snowfields in Holarctic mountain ranges (0–5000 m. above sea level). Morphological measurements, including pronotal ratio (widest point divided by the base), elytral length, elytral ratio (length divided by width), antennal scape length, and pronotal and elytral shape (Fourier decomposition), were used in this study, in conjunction with measurements of habitat use habitat where specimens were collected (1970‐2021). Morphological variation was examined in relation to habitat use and phylogenetic relatedness, and morphological trait evolution was tested for convergence. Ecomorphological traits are evolving slower than expected under a null model of Brownian motion evolution. *Nebria* species cluster in multivariate morphospace according to relatedness, but habitat use and relatedness together are the best predictors of morphological variation. There is evidence for convergence in riparian species based on morphologicy alone, and additional evidence for morphological convergence in riparian and alpine species when phylogenetic distance is considered. In species assemblages of *Nebria*, we found evidence of rapid diversification followed by a slow rate of ecomorphological evolution, with convergent evolution playing a significant role in shaping trait‐habitat use patterns and niche acquisition.

## Graphical abstract

A critical step toward uncovering generalizable patterns of phenotype‐niche relationships is understanding how functional traits have evolved as species occupy new habitats. By integrating a molecular phylogeny, habitat use, and morphometrics, this study aimed to understand the role of ancestry and convergent evolution in ecomorphological trait evolution within a Holarctic, montane ground beetle community. We found evidence of rapid diversification followed by a slow rate of ecomorphological evolution, with convergent evolution playing a significant role in shaping trait‐habitat use patterns and niche acquisition.

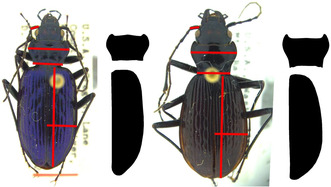

## 1 Introduction

A major goal in macroevolution is to discover generalizable patterns of phenotypic evolution and niche evolution (Weber et al. 2017). Examining the evolutionary processes that underlie adaptive radiations provides critical insight into the rules shaping niche diversification, through phenomena such as convergent evolution. Convergent evolution often arises in species radiations as a result of the interaction between traits and environment, where different species evolve similar traits in response to resource availability and/or habitat‐based performance (terHost et al. 2010). One common pattern is for physical form, morphology, to evolve predictably to environmental conditions (Vidal‐García and Keogh 2015). Morphological similarity can also arise due to ancestral constraint, where species remain similar to one another due to shared genes and similar selective pressures (Losos 2011). While these processes can be difficult to distinguish in a recent species radiation, if the relationship between functional traits (D'Andrea and Ostling 2016) and niche (Elton 1946) can be explained by patterned, repeated development of traits across species, and this pattern cannot be explained by recent shared evolutionary history, convergent evolution may be occurring (Pigot et al. 2020). Adaptive radiations present a useful opportunity to examine the role of convergent evolution in trait‐niche patterns. Recent shared ancestry among taxa, paired with ecological diversity, makes these dynamics easily testable. Adaptive radiations are relatively rapidly diversifying species groups who subsequently occupy a variety of niches (Schluter 2000). Numerous studies have documented how ecological opportunity drives adaptive radiations, for example in finches (Grant and Grant 2002), cichlids (Sturmbauer 1998), *Anolis* spp. lizards (Butler and King 2004), and spiders (Gillespie et al. 2018), among other taxa. Separately, other studies have looked for repeated ecological trait patterns in nature, such as size ratios among closely competing beetles, ultimately leading to competitive exclusion of similar sizes (Hutchinson 1959; Brandl and Topp 1985) or other functional morphological traits (Stayton 2006; Pigot et al. 2020) which may indicate niche partitioning or competitive exclusion. Ecological specialization is a key component of adaptive radiations, and is a critical requisite for examining processes influencing trait‐niche relationships. In this study, we examined the role of convergent evolution in ecological specialization within a community of montane ground beetles (Carabidae: *Nebria*).

This Holarctic genus represents a relatively recent adaptive radiation (Slatyer and Schoville 2016; Kavanaugh et al. 2021). Species of *Nebria* are ecological specialists and occur in riparian and snowfield habitats across a wide range in elevation (0–5000 m. above sea level). In these habitats, species occupy sequentially overlapping, but statistically discernable elevational ranges. *Nebria* species are known to have close associations with habitat type (Kavanaugh 1979; Schoville et al. 2012). In these species assemblages, environmental filtering appears to be the dominant ecological process influencing species distributions (Schat et al. 2024). The habitats used by species of *Nebria* species vary in composition from small sandy riverbeds, pebble‐bound streams, rocky rivers, boulder‐entrenched rivers, to talus snowfields. These habitats occur in isolated mountains across the Northern Hemisphere, where independent lineages of these species have evolved due to geological barriers (Kavanaugh et al. 2011). While adaptive radiations have been studied in many different environments, few examine cold temperate communities where species richness is typically low. The genus *Nebria* is an interesting example of a taxon that has high species richness in these habitats.

One interesting way to test the influence of convergent evolution in ecological specialization is to examine the evolutionary trends of ecologically important morphological traits. In many species, ecomorphological traits evolve as a result of niche expansion or contraction (D'Andrea and Ostling 2016), making functional traits ideal characteristics for testing hypotheses of repeated patterns in niche acquisition. Body size and pronotal width ratio are both ecomorphological traits that can reliably predict habitat use of the 12 species of *Nebria* present on Mt. Rainier in the North American Cascades Range (Schat et al. 2024), as well as *Nebria* species in Russia (pers. comm. R.Y. Dudko). Pronotal and elytral shapes are traits related to habitat usage and locomotion in carabid species, and are known to vary across environmental gradients (Thiele 1977). Body size impacts the movement of species in their microhabitat (e.g., their ability to move through gaps in talus rock piles) (Kaspari and Weiser 1999) and elytral length is a commonly used proxy for body size, which is known to predict habitat use in ground beetles in general (Schirmel et al. 2015; Schat et al. 2022) and even intraspecies larval viability (Nelemans 1987). According to Bergmann's Law, body size may be a useful signal of alpine specialization, although the consistency of this pattern varies across insects (Shelomi 2012). Pronotal shape influences forelimb mobility, significantly impacting how these geophilic species navigate substrate texture and their rate of movement (Laparie et al. 2013; Weiser and Kaspari 2006; Talarico et al. 2007; Jachertz et al. 2019). *Nebria* species are mostly flightless (Kavanaugh 1978; Schoville et al. 2012), and even macropterous species very rarely fly (Nelemans 1987). *Nebria* species reproduce, forage, and seek refuge in and under substrates, and so their ability to traverse various grain sizes in riparian habitats or consistent grain sizes (snow) in alpine communities would impact their survival. In addition, forelimb mobility impacts navigation across substrates of varying grain size, and the homogeneity of snowfield substrate may result in less variation of this trait. *Nebria* species specialize on specific habitat types (Gereben 1995), and exploring the evolution of ecomorphological traits in the context of habitat use can provide insight into the evolutionary processes behind this ecological specialization.

Here, we will examine whether ecomorphological traits converge within riparian and alpine species assemblages where constituent species of *Nebria* vary. Alpine habitats are found above the tree line, while riparian habitats are found below the tree line. This abrupt delineation in habitat type will provide contrasting environments across which ecomorphological convergence can be clearly examined. In habitats where *Nebria* species are found, alpine habitat use corresponds to rocky environments associated with snowfields and seep habitats, while non‐alpine habitat use is closely tied to highly variable riparian environments. Species of *Nebria* are crepuscular and seek refuge under snow, talus, gravel, and sand during the day. Gross body proportions, such as the ratio of pronotal size to elytral size are expected to influence species' movement through these habitats. We expected that larger species with less square pronota would be more closely associated with alpine habitats (Schat et al. 2024). We hypothesize that the evolution of ecomorphological traits will demonstrate convergence with relation to habitat specialization. That is, riparian species' traits will cluster with one another, and will differ from alpine traits, regardless of the relatedness of alpine habitat specialists or riparian habitat specialists. Similarly, alpine species' traits will be more similar to one another than to riparian species, regardless of ancestry. Repeated evolution of similar forms in similar environments, across distantly related species in a phylogeny would provide strong evidence for convergent evolution (Losos 2011). Because morphological similarity among taxa may occur simply due to random processes, we examine the rate of evolution of ecomorphological traits as well. Traits evolving significantly faster or slower than a random walk are likely experiencing natural selection, and so the rate of evolution of ecomorphological traits can indicate underlying processes influencing their variation. In this study, we (1) determine the rate of evolution of ecomorphological traits, (2) test whether habitat use or evolutionary history predicts variation in traits, and (3) test whether species have converged on habitat‐specific morphotypes.

## 2 Methods

### 2.1 Focal Taxa

**FIGURE 1:**
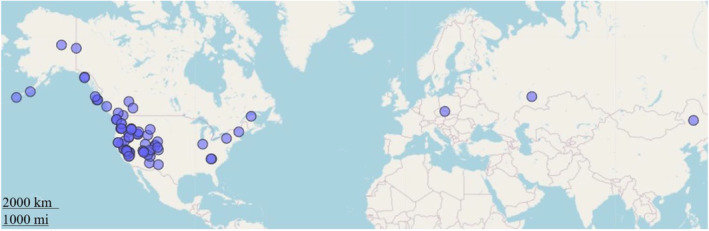
Map of locations where specimens of species of *Nebria* used in this study were collected. Each point represents a site where multiple specimens of any species present were collected.

Species of *Nebria* have a Holarctic distribution and occupy primarily talus snowfield and riparian habitats in montane ecosystems (Kavanaugh 1978; Schoville et al. 2012; Kavanaugh et al. 2021; Schat et al. 2024). This study focuses on species of *Nebria* within their Nearctic range due to the accessibility of specimens for morphometric analysis, but it includes Palearctic species of *Nebria* as well (Figure 1, figure made using the R packages “mapview” (Appelhans et al. 2022), “sf” (Pebesma 2018; Pebesma and Bivand 2023), and “tidyverse” (Wickham et al. 2019)). Specimens that were photographed for morphometric analysis are housed within the California Academy of Sciences. Across 79 species, an average of 17 specimens per species were used in this study (mean = 17.4, sd = 4.00, see sampling details in Appendix S1, Table S1).

### 2.2 Morphometrics

**FIGURE 2:**
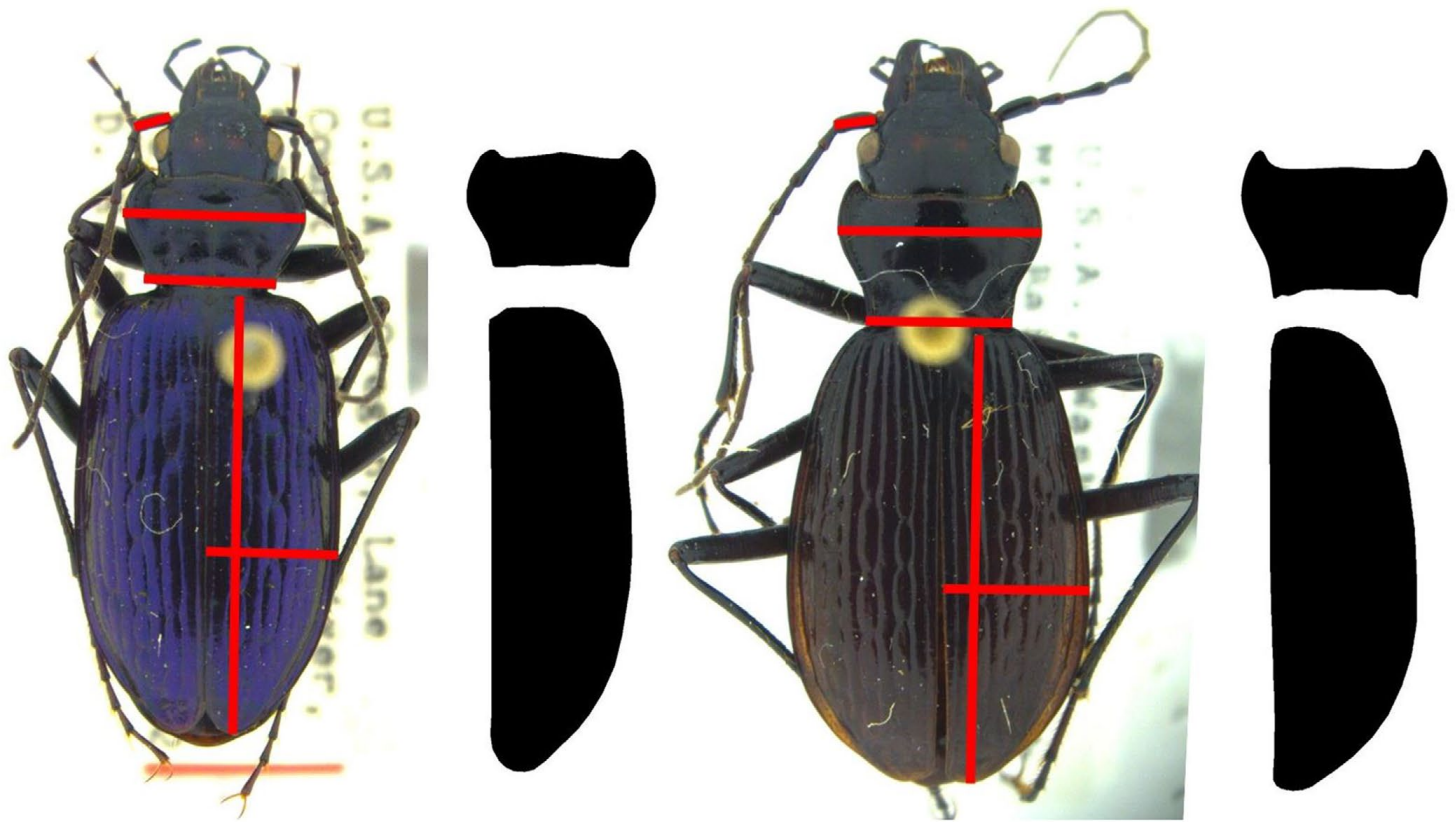
Specimens of *Nebria piperi* (left), a riparian species, and *N. vandykei* (right), an alpine species (PC: Schat et al. 2022). Images are not to scale. Red lines indicate where measurements were taken to assess linearly measured morphological traits, including elytral length, elytral ratio (length divided by width), pronotal width ratio (widest distance divided by the base), and antennal scape length. Silhouettes are examples of pronotal and elytral shape silhouettes created in Inkscape.

Photographs of these specimens were taken using a Leica camera mounted to a dissecting scope (Leica Camera AG 2020). Raw images of the pronotum and elytra were processed to generate silhouettes for Fourier decomposition analysis. These silhouettes were created using the software Inkscape (Inkscape 2020). Antennal scape length, elytral length (along elytral suture), ratio of the elytral length to the elytral width (elytral width ratio), and ratio of the pronotum at the widest point to the base (pronotal width ratio) were measured on each specimen using the software ImageJ (Schneider et al. 2012) (Figure 2). These features are known to vary across species of *Nebria* and predict habitat usage in the northern Cascades Range (Schat et al. 2024). We did not scale antennal scape length by body size, as is common in entomological studies; in previous work, it has been shown that antennal scape length is orthogonal to body size (Schat et al. 2024), and so this step would not add additional information about allometric scaling. Antennal scape shape will be used to compare the rate and pattern of morphological trait evolution, as it is not expected to vary in relation to habitat type, yet frequently diverges among closely related species (Weng et al. 2020).

All statistical analyses in this study were performed using R v4.2.0.1 (R Core Team 2023). A multivariate analysis of variance (MANOVA) was used to assess whether these traits vary across the species of *Nebria* analyzed in this study. The Student's T‐test was used to assess whether these traits vary between sexes. Fourier decomposition was used to extract shape features from silhouettes (Figure 2) of elytra and pronota using the R package “Momocs” (Bonhomme et al. 2014), while the packge “tibble” was used to organize data tables (Wickham et al. 2019).

### 2.3 Habitat Use

Locality data from specimen labels were used to estimate habitat and local climate conditions. When available, GPS coordinates were used to directly extract local climatic information. Otherwise, approximate locations were determined based on specimen labels. Google Earth (Google Maps 2021) was used to approximate the substrate type, elevation, and coordinates (when not directly stated on labels) where beetles were collected. The online database WorldClim (Fick and Hijmans 2017) was used to estimate mean annual ground temperature (˚C), maximum annual ground temperature (˚C), minimum annual ground temperature (˚C), mean annual solar radiation (kJ/m^2^/day), mean annual vapor pressure (kPa), annual precipitation volume (mm), and mean annual wind speed (m/s) for each site. Estimates for each of these variables are accurate to the nearest 30 arcseconds (~1 km^2^). Habitat variables were examined for cross‐correlation and were only included in further analysis if they were not strongly correlated with one another (if the absolute value of Pearson's *r* < 0.75).

We tested for morphological convergence using two thresholds for “alpine” (above the tree line) and “riparian” (below the tree line) habitats. The tree line in montane ecosystems represents a stark demarcation of alpine habitat. In systems where environmental filtering is dominant, such contrasting, close‐proximity habitats can be a useful setting in which to test for ecomorphological convergence based on habitat type. Körner and Paulsen (2004) found that alpine habitats in temperate zones typically have a seasonal (growing season) mean ground temperature of 7–8˚C, while subarctic and boreal alpine habitats have a seasonal mean ground temperature of 6–7˚C. This thermal threshold corresponds to the tree line in these habitats, with alpine habitats found above the tree line. While the species used in this study could be categorized as temperate, boreal, or subarctic based on their locality data, some species analyzed in this study occupy ranges that span multiple biomes. We used thresholds of mean ground temperature 6˚C or colder, as well as 7˚C or colder to define alpine habitats. Convergence under each of these thermal conditions was assessed separately.

### 2.4 Phylogenetic Relatedness

**FIGURE 3:**
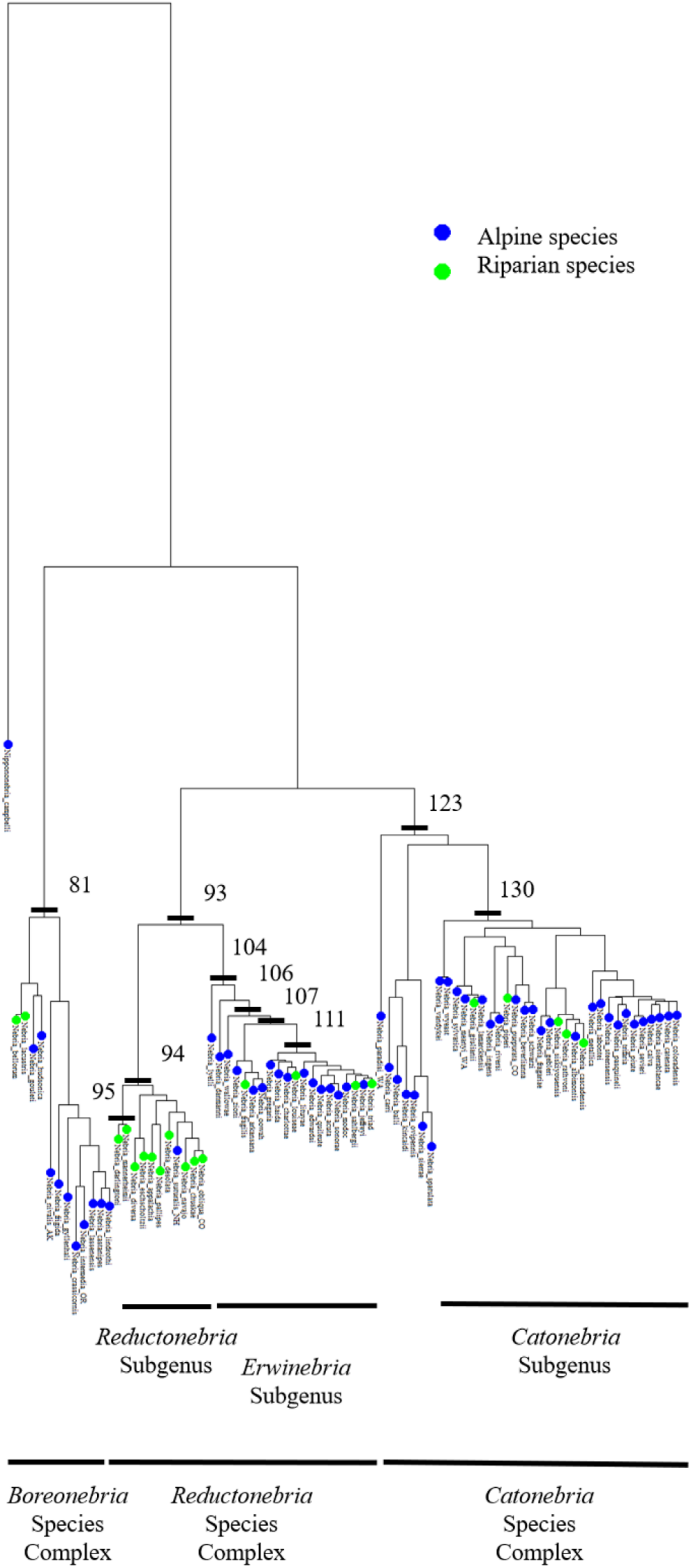
Phylogeny of species of *Nebria* used in this study based on the multilocus Maximum Likelihood phylogeny found in Kavanaugh et al. (2021), rooted with the outgroup *Nippononebria campbelli* . Colors indicate whether species were collected in alpine or riparian habitats based on a threshold mean ground temperature of 6˚C. Bars indicate nodes at which the evolutionary rate of one or more traits changed. Nodes 81, 93, and 123 correspond to the species complexes *Boreonebria*, *Reductonebria*, and *Catonebria*, respectively. Nodes 130, 104, and 94 correspond to the subgenera *Catonebria*, *Erwinebria*, and *Reductonebria*, respectively. Elytral length evolution slowed at nodes 95, 111, and 123. Elytral ratio evolution slowed at nodes 104 and 130. Pronotal ratio evolution slowed at nodes 111 and 130. Antennal scape length evolution slowed at nodes 94, 111, and 123. Pronotal shape evolution slowed at nodes 107 and 130. Elytral shape evolution slowed at nodes 106 and 130.

A multi‐locus molecular phylogeny by Kavanaugh et al. (2021) was used to identify the role of phylogenetic relatedness in trait variation (Figure 3). This phylogeny was based on maximum likelihood estimates of relatedness using a concatenated dataset that included the nuclear ribosomal gene 28S, nuclear protein coding genes CAD2, PEPCK, Topo, and wg, and the mitochondrial genes 16S‐ND1, COI BC, and COI PJ (Kavanaugh et al. 2021). Nodes with less than 90% bootstrap support were collapsed. While the full phylogeny is rooted with numerous outgroups and other *Nebria* taxa, we pruned the tree to contain only our focal taxa and one outgroup species— *Nippononebria campbelli* (a member of the same tribe: Carabidae: Nebriini).

### 2.5 Statistical Analysis of Morphometrics, Habitat Use, and Phylogenetic Relatedness

As an initial analysis, k‐means cluster analysis was used to discern if species cluster in morphospace, independent of relatedness. Linearly measured traits (elytral length, elytral ratio, pronotal ratio, and antennal scape length), elytral shape, and pronotal shape were each analyzed separately. The number of clusters was determined based on minimizing the within‐group sum of squared distance from k centroids. In a separate analysis, regression was used to assess the ability of habitat use and phylogenetic relatedness to predict morphological variation in species of *Nebria*. Habitat features and relatedness were assessed separately in two different models, and together in the same model, for a total of three sets of regression models. Based on a power analysis using the R package “simmr” (Green and Macleod 2015), a sample size of approximately 15 individuals per species is needed to detect an effect size of 0.05 with a power of 80% and α = 0.05 in a generalized mixed model. This sample size was achieved in nearly all species. In models that included relatedness as a predictor, the phylogeny of species of *Nebria* was converted to a correlation matrix of relatedness among species, assuming Brownian motion as a model for the rate of evolution. In models that included habitat features, latitude and elevation were included as fixed effects.

These models used a correlation matrix of linearly measured traits (antennal scape, elytral length, elytral ratio, pronotal ratio), or a correlation of pronotal shape features, or a correlation matrix of elytral shape features as response variables. Likelihood ratio tests were used to compare models which included relatedness and/or habitat characteristics as predictors.

### 2.6 Convergence and Trait Evolution

Under a null model of Brownian motion, trait evolution follows a random walk that is constrained by recent ancestry, with more closely related species being more similar and variation increasing linearly with evolutionary distance (Pagel 1997; Ives and Zhu 2006; Ives 2018). Traits undergoing directional selection will likely experience faster rates of change than expected by random chance (Clegg et al. 2002), especially in the early stages of this process. Traits undergoing stabilizing selection will likely experience slower rates of change than expected by random chance (Wojcieszek and Simmons 2012, but see Lemos et al. 2001). The function search. shift in the R package “RRphylo” (Castiglione et al. 2019) was used to search for shifts in the evolutionary rate of ecomorphological traits across species lineages. It uses the results from a phylogenetic ridge regression (using the function RRphylo in the same package). Ridge regression works similarly to regression, although assumes that some data are highly correlated, as would occur among taxa in a phylogeny. Phylogenetic ridge regression allows this assumption of correlated to be adjusted according to the topology of the phylogeny. The search. shift function then estimates clade‐specific evolutionary rates which explain this relationship. In this study, the minimum clade size was set to one branch. We also tested differential rates of ecomorphological trait evolution between alpine and riparian species. Using the “sparse” argument for status. type, instead of “clade”, both “alpine” and, separately “riparian” was tested for differential rate of evolution from the rest of the tree. More than half the species are alpine, and so testing for both specializations as the “state” of interest may yield different results. Early burst (Simpson 1944; Schluter 2000) and Uhlenbeck and Ornstein (1930) methods are commonly used to assess convergence upon trait optima in phylogenies; however, these approaches often result in model overfitting in multivariate datasets such as these and often favor more complex models, even when data is generated under simulations of Brownian motion (Adams and Collyer 2018). Instead, it is recommended that regression and comparison to models of Brownian motion be used to assess the evolution of traits in multivariate datasets (Adams and Collyer 2018).

Morphological convergence toward habitat specific morphotypes was assessed among species of *Nebria* assuming an alpine mean ground temperature of 6˚C, and separately, 7˚C. To assess convergence upon alpine or riparian morphotypes, two methods were used.

First, several metrics of convergence were calculated (C1‐4) based on the methods described in Stayton (2015). C1 tests for evidence of convergence by calculating: 1‐D_tip_/D_max_. Here, D_tip_ represents the greatest phenotypic distance between species in multivariate space (Euclidean or Procrustes, whichever is larger), and D_max_ represents the greatest phylogenetic distance between species (branch length between taxa) (Stayton 2015). This allows for phenotypic change to be assessed in the context of the general evolutionary rate of taxa, preventing rapidly (or very slowly) evolving taxa from skewing results. Values for C1 range between 0 and 1, with a larger value indicating greater convergence. C2 estimates the proportion of phenotypic distance “closed” by convergent evolution and is calculated by: D_max_—D_tip_. C3 indicates the proportion of morphological change within a lineage that can be explained by convergence and is calculated by: C2/L_tot.lineage_. The value L_tot.lineage_ is the total morphological change in that lineage from their ancestral state. Lastly, C4 indicates the proportion of morphological change within a clade that can be explained by convergence and is calculated by: C2/L_tot.clade_. The value L_tot.clade_ is the total morphological change in that clade from the ancestral state (Stayton 2015). These calculations were performed using the function calcConv in the R package “convevol” (Brightly and Stayton 2023). A strength of these metrics is their ability to detect pattern‐based convergence of morphological traits, regardless of the context under which they have converged. However, these comparisons are not transferrable across taxonomic groups and must be interpreted only within the designated taxa. The significance of these values was assessed using permutation tests. C1‐4 metrics have the risk of identifying divergent, outlying individuals or taxa as convergent, when they are in fact divergent, especially if they are distantly related (Grossnickle et al. 2024). In a closely related species set, such as this, this bias is less likely to be consequential. Using additional methods, such as k‐means clustering, can identify inconsistencies in our analyses of convergence, reducing the possibility of drawing misleading conclusions.

Second, species of *Nebria* were plotted in multivariate morphospace. The angle between pairs of species were used to approximate the similarity between species, with a smaller angle indicating greater similarity (Sansalone et al. 2020). Using the search.conv function in the R package “RRphylo” (Castiglione et al. 2018), convergence upon alpine or riparian morphotypes was assessed. If angles between species that occupy the same habitat type are smaller than the average angle among all species (if alpine species cluster with other alpine species in morphospace, for instance), then species may be converging on a habitat‐specific morphotype. Additionally, if no convergence is occurring, the angle between species should increase with time since divergence (Sansalone et al. 2020). The angle between species of the same habitat category, and angle between species per unit time was used to quantify convergence. The significance of these findings was assessed using permutation tests.

## 3 Results

### 3.1 Morphometrics

We first confirmed whether *Nebria* species could be distinguished based on our measured morphological trait data. Based on MANOVA tests, elytral ratio (*F*_78,1295_ = 13.21, *p* < 0.001), pronotal ratio (*F*_78,1295_ = 41.39, *p* < 0.001), antennal scape length (*F*_78,1295_ = 135.9, *p* < 0.001), and elytral length (*F*_78,1295_ = 132.1, *p* < 0.001) all vary significantly among species of *Nebria*. Only elytral length varies by sex (*t*_1371_ = 2.96, *p* = 3•10^−3^). While statistically discernable, the distributions of elytral length of males and females are highly overlapping and the effect is small compared to elytral variation among species (Appendix S1, Figure S1), and so males and females were grouped together by species in further analyses. All linearly measured morphological traits are approximately normally distributed across species of *Nebria* (Appendix S1, Figure S2).

### 3.2 Morphometrics, Habitat Use, and Phylogenetic Relatedness

**FIGURE 4:**
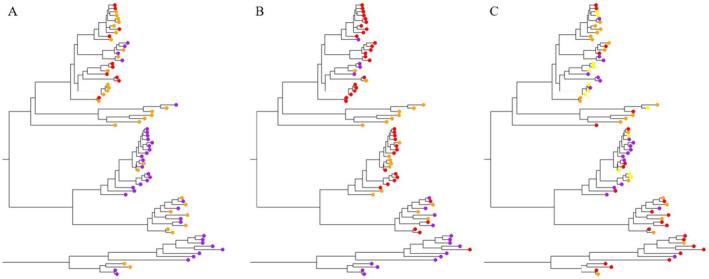
Phylogeny of species of *Nebria* used in this study based on the multilocus Maximum Likelihood phylogeny found in Kavanaugh et al. (2021). Images of the phylogenies were trimmed for visualization, but the topology is unchanged. K‐means clustering was used to assess how species cluster in morphospace. Based on the within‐group sum of squared distance from centroids, linearly measured traits together formed three clusters (A), pronotal shape features formed three clusters (B), and elytral shape features formed four clusters in morphospace (C). Different colors represent different clusters in each panel. In panels A and B, red, orange, and purple tips are used to indicate whether taxa belong to cluster 1, 2, or 3 respectively. In panel C, red, orange, purple, and yellow tips are used to indicate whether taxa belong to cluster 1, 2, 3, or 4 respectively.

We next examined whether habitat features and phylogenetic relatedness account for variation in morphological traits among species of *Nebria*. Habitat characteristics where species of *Nebria* were collected are approximately normally distributed across species (Appendix S1, Figure S3). Based on the within‐group sum of squared distance from centroids, elytral shape features formed four clusters in morphospace, pronotal shape features formed three clusters, and linearly measured traits together formed three clusters. MANOVAs showed that none of these clusters correspond to variation in habitat use (Appendix S1, Figures S4–S6), including variation in elevation, latitude, longitude, solar radiation, vapor pressure, mean precipitation, wind speed, substrate type, nor mean, maximum, or minimum ground temperature. However, after converting the phylogeny to a correlation matrix, analyses of variance (ANOVA) showed that relatedness explains the clustering of species in multivariate morphospace (elytral shape features: *F*_3,6237_ = 85.4, *p* < 0.001; pronotal shape features: *F*_2,6238_ = 363, *p* < 0.001; linearly measured traits: *F*_2,6238_ = 102, *p* < 0.001) (Figure 4), indicating ancestral constraint as a predictive factor in morphological similarity. Tukey HSD tests showed that all clusters were statistically discernable from one another in elytral shape, pronotal shape, and the morphospace of linearly measured traits.

Linear regression was used to assess how much variation in morphological traits could be explained by phylogenetic relatedness and habitat use. Models that included both relatedness and habitat use explained a small, but significant portion of variation in morphological traits (linearly measured traits: adjusted *R* ^2^ = 0.0546, *p* < 0.001; pronotal shape: adjusted *R* ^2^ = 0.121, *p* < 0.001; elytral shape: adjusted *R* ^2^ = 0.0738, *p* < 0.001). Based on likelihood ratio tests, models that included both relatedness and habitat use outperformed models that only included relatedness (linear traits: *X*_6_^2^ = 135, p < 0.001; pronotal shape: *X*_6_^2^ = 203, *p* < 0.001; elytral shape: *X*_6_^2^ = 379, *p* < 0.001) or only habitat use (linear traits: *X*_5_^2^ = 57.3, *p* < 0.001; pronotal shape: *X*_5_^2^ = 323, *p* < 0.001; elytral shape: *X*_5_^2^ = 262, *p* < 0.001) as predictors.

### 3.3 Trait Evolution and Tests for Convergence

We next assessed whether morphological traits showed differential rates of evolution and evidence for convergence upon habitat type. Following a Bonferroni correction for multiple comparisons, we found that the evolutionary rate of all morphological traits, as well as antennal scape length, decreases across species lineages (Figure 3). Elytral length evolution slowed at nodes 95 (rate Δ: −4.95•10^−3^, *p* = 0.02), 111 (rate Δ: 5.27•10^−3^, *p* = 0.01), and 123 (rate Δ: 7.43•10^−3^, *p* = 0.01). Elytral ratio evolution slowed at nodes 104 (rate Δ: 3.88•10^−4^, *p* = 0.01) and 130 (rate Δ: 3.79•10^−4^, *p* = 0.01). Pronotal ratio evolution slowed at nodes 111 (rate Δ: 6.80•10^−4^, *p* = 0.01) and 130 (rate Δ: 8.40•10^−4^, *p* = 0.01). Antennal scape length evolution slowed at nodes 94 (rate Δ: 4.19•10^−2^, *p* = 0.03), 111 (rate Δ: 4.51•10^−2^, *p* = 0.01), and 123 (rate Δ: 6.45•10^−2^, *p* = 0.01). Pronotal shape evolution slowed at nodes 107 (rate Δ: 3.44•10^−4^, p = 0.01) and 130 (rate Δ: 3.90•10^−4^, *p* = 0.01). Elytral shape evolution slowed at nodes 106 (rate Δ: 2.36•10^−4^, *p* = 0.01) and 130 (rate Δ: 2.67•10^−4^, *p* = 0.01). This indicates that these traits are undergoing stabilizing selection.

Nodes 81, 93, and 123 correspond to species complexes *Boreonebria*, *Reductonebria*, and *Catonebria*, respectively. Node 123, the species complex *Catonebria*, is the location of shifts in elytral length and antennal length rates. The subgenus *Catonebria* diverges at node 130, corresponding to shifts evolutionary rate of elytral ratio, pronotal ratio, pronotal shape, and elytral shape. This species complex includes some of the largest species of *Nebria* known. Within the *Reductonebria* species complex, there are two clades—the subgenus *Reductonebria*, which diverges at node 94, and the subgenus *Erwinebria*, which diverges at node 104. With the divergence of *Reductonebria*, antennal scape length evolution slows, and with the divergence of the subgenus *Erwinebria*, elytral ratio evolution slows. The subgenus *Reductonebria* is dominated by riparian species (90.9% of species in this clade) whereas the subgenus *Erwinebria* is dominated by alpine species (80% of species in this clade). These shifts in ecomorphological trait evolution at these nodes may correspond to shifts in habitat use.

Morphological convergence for alpine and riparian morphotypes was assessed among species of *Nebria*, assuming an alpine mean ground temperature of 6˚C, and separately, 7˚C. Based on the metrics described in Stayton (2015), elytral shape appears to be converging among riparian species. This is consistent whether using a 6˚C (C1 = 0.268, *p* = 0.009; C2 = 0.000423, *p* = 0.012; C3 = 0.111, *p* = 0.004) or 7˚C (C1 = 0.256, *p* = 0.025; C2 = 0.000409, *p* = 0.027; C3 = 0.108, *p* = 0.015) threshold of mean ground temperature to categorize habitat type (Table 1). Pronotal shape also appears to be converging among riparian species, relative to other species in their clade (6˚C: C4 = 0.000521, *p* = 0.0003; 7˚C: C4 = 0.000496, *p* = 0.008) (Table 1). Linearly measured morphological traits appear to be converging among alpine species, relative to other species in their clade (6˚C: C4 = 0.00578, *p* = 0.055; 7˚C: C4 = 0.00546, p = 0.05) (Table 1).

**TABLE 1:** Extent of convergence on a riparian and alpine morphotypes across species of *Nebria* based on C1‐4 metrics as described in Stayton (2015). Riparian habitats are those which have a mean ground temperature of > 6˚C—7˚C. Alpine habitats are those which have a mean ground temperature of 6˚C—7˚C or less.

	C1	C2	C3	C4
Riparian Morphotype (> 6˚C)				
Linearly Measured Traits	0.375	0.615	0.168	0.00168
Pronotal Shape	0.165	0.000534	0.0675	0.000521*
Elytral Shape	0.268*	0.000423*	0.111*	0.000710
Riparian Morphotype (> 7˚C)				
Linearly Measured Traits	0.371	0.576	0.167	0.00174
Pronotal Shape	0.167	0.000547	0.0692	0.000496*
Elytral Shape	0.256*	0.000409*	0.108*	0.000835
Alpine Morphotype (≤ 6˚C)				
Linearly Measured Traits	0.316	0.604	0.167	0.00578*
Pronotal Shape	0.173	0.000566	0.0672	0.0000971
Elytral Shape	0.175	0.000261	0.0720	0.00146
Alpine Morphotype (≤ 7˚C)				
Linearly Measured Traits	0.321	0.620	0.169	0.00546*
Pronotal Shape	0.174	0.000563	0.169	0.00546
Elytral Shape	0.176	0.176*	0.0719	0.00140

* Statistical significance (*p* < 0.05) was calculated from permutation tests.

** *p* < 0.10.

Based on proximity in multivariate morphospace, riparian species cluster together based on linear measurements of morphology (antennal scape length, elytral length, elytral ratio, and pronotal ratio) (6˚C: θ = 2.49, *p* = 0.058; 7˚C: θ = 2.55, *p* = 0.072) (Table 2). They do not cluster in morphospace based on pronotal shape (6˚C: θ = 2.28, *p* = 0.456; 7˚C: θ = 2.31, *p* = 0.532) or elytral shape (6˚C: θ = 1.56, *p* = 0.380; 7˚C: θ = 1.60, *p* = 0.498). Alpine species do not cluster together in multivariate morphospace whether analyzing linearly measured traits (6˚C: θ = 3.25, *p* = 0.955; 7˚C: θ = 3.23, *p* = 0.945), pronotal shape (6˚C: θ = 2.30, *p* = 0.470; 7˚C: θ = 2.28, *p* = 0.423), or elytral shape (6˚C: θ = 1.57, *p* = 0.258; 7˚C: θ = 1.57, *p* = 0.230) (Table 2).

**TABLE 2:** Angular distance (θ) between riparian and alpine species in multivariate morphospace. Riparian habitats are those which have a mean ground temperature of > 6˚C–7˚C. Alpine habitats are those which have a mean ground temperature of 6˚C–7˚C or less.

	θ	θ adjusted by time
Riparian Morphotype (> 6˚C)		
Linearly Measured Traits	2.49*	77.3
Pronotal Shape	2.28	64.5
Elytral Shape	1.56	58.9
Riparian Morphotype (> 7˚C)		
Linearly Measured Traits	2.55**	80.4
Pronotal Shape	2.31	66.6
Elytral Shape	1.60	62.3
Alpine Morphotype (≤ 6˚C)		
Linearly Measured Traits	3.25	66.0
Pronotal Shape	2.30	52.2
Elytral Shape	1.57	40.1
Alpine Morphotype (≤ 7˚C)		
Linearly Measured Traits	3.23	66.3
Pronotal Shape	2.28	51.9
Elytral Shape	1.57	40.1

* Statistically significant values (*p* < 0.05).

** *p* < 0.10.

## 4 Discussion

### 4.1 Convergent Evolution of Morphospace Among Species of Nebria

The similarity of ecomorphological traits among riparian species suggests that there is convergence upon morphotypes. Evidence is much stronger for the riparian morphotype, and is supported by similarity of linearly measured traits, elytral length, elytral ratio, pronotal width ratio, and scape length. When accounting for phylogenetic distance between species, elytral shape appears to be undergoing convergence in riparian species, as is pronotal shape when compared to non‐riparian species in the clade. When accounting for phylogenetic distance between species, only linearly measured traits appear to be converging among the alpine morphotype.

A small, but significant proportion of the variation in ecomorphological traits is best predicted by relatedness and habitat use together, but the clusters that species form in multivariate morphospace are only predicted by relatedness. This apparent discrepancy may be explained by the way habitat features are included in these analyses. The habitat features used in this study measure habitat states to the nearest ~1 km^2^, whereas species of *Nebria* tend to specialize on habitat *and* microhabitat features (Schat et al. 2024). A linear combination of course‐grain habitat features may not be enough to explain morphospace partitioning, and finer scale analyses of microhabitat features could provide more explanatory power as to how communities partition their habitat.

All ecomorphological traits and antennal scape length are evolving slower than expected under Brownian motion evolution, suggesting that these traits are undergoing stabilizing selection. Directional selection and disruptive selection would increase the rate at which traits evolve. Balancing selection could also result in a slowing of evolutionary change as stabilizing selection does, but balancing selection would also result in greater trait variability, whereas stabilizing selection also reduces trait variation. For these reasons, we conclude that stabilizing seems to be the most likely explanation for the reduction in evolutionary rate of change and trait variability, as assessed by species clustering in morphospace. The evolutionary rates of all measured traits shift several times throughout the evolutionary history of species used in this study. Some of these shifts appear to coincide with changes in habitat specialization, while others coincide with divergence of the specific clades. Elytral length is often used as a proxy for body size (Sukhodolskaya 2013; Luzyanin et al. 2022), which along with pronotal ratio and elytral length, have been shown to be important predictors of habitat use (Schat et al. 2022; Schat et al. 2024). Within adaptive radiations, early rapid diversification, followed by decreases in trait evolution can indicate convergence upon ecological morphotypes (Sansalone et al. 2020).

### 4.2 Habitat Use in Montane Environments

Montane environments possess ecotones spanning elevations and latitudes, and species of *Nebria* are known to specialize in niches defined by ecotones in these habitats (Grinnell 1917; Kavanaugh 1979, p35‐57; Gobbi et al. 2007; Schoville et al. 2012; Slatyer and Schoville 2016; Schat et al. 2024). The convergence observed in elytral shape and pronotal shape suggests that the gestalt of these morphotypes may be consistent with riparian or alpine specialization, such as having ovoid (alpine) or angular (riparian) elytra. Within riparian or alpine communities, further habitat specialization at the microhabitat scale may explain the rate of evolution of body size (elytral length) and body proportions (elytral ratio and pronotal ratio). Morphological traits, specifically body size and pronotal width ratio, have been shown to predict habitat and microhabitat use in northern Cascades Range environments (Schat et al. 2024). In addition, the scaling of morphological traits is known to vary with elevation (Sukhodolskaya and Ananina 2015). In alpine habitats, many animal species possess more compact morphology—thicker and shorter limbs, larger bodies (Bergmann's Law). It is possible that the ovoid shape of the elytra in these species indicates a shift toward a more compact morphology, although this requires further investigation. The consistency of these relationships in these species assemblages, regardless of local species composition, suggests that environmental filtering dictates species persistence and may have resulted in similar morphotypes within separate, but similar habitat types. Convergence upon ecologically consistent morphotypes suggests strong environmental filtering. Environmental filtering also impacts species establishment within a community, and it is likely that these processes are happening simultaneously. Environmental filtering is known to be a dominant factor in predicting species diversity in alpine and montane habitats (Janzen 1967; Machac et al. 2011), including in ground beetles (Gobbi et al. 2007). The convergent processes indicated in this study help explain patterns of morphological evolution previously documented in closely related *Nebria* taxa (Weng et al. 2020; Schoville et al. 2023).

In species assemblages with strong environmental filtering, phenotypic similarity among coexisting species is expected (Cavender‐Bares et al. 2006). When these assemblages are composed of closely related species, this phenotypic similarity can be due to relatedness. However, in clades with high diversification rates, similarity may be the result of convergent evolution. This appears to be the case in *Nebria* species. Convergence upon morphotypes related to niche has been seen in other ground beetle communities (Ingerson‐Mahar 2014; Maddison et al. 2019; Baulechner et al. 2020). Morphology is a known predictor of habitat use (Maddison and Maruyama 2019), resource consumption (Konuma and Chiba 2007), and extinction risk (Nolte et al. 2019) among ground beetles. Elytral shape, pronotal shape, and antennal scape length are known to vary across species within ground beetle communities and predict habitat use (Ribera et al. 1999). The consistent relationships between habitat use, species diversity, and functional diversity among ground beetles is what has made them such reliable bioindicators of changes in habitat (Koivula et al. 2002) and ecosystem diversity (Pakeman and Stockan 2014).

### 4.3 Glacial Cycles and Habitat Partitioning

As habitat specialists, species of *Nebria* respond to climatic cycles by chasing their climatic niche across elevations (Schoville et al. 2012). In some Palearctic communities of these species, the age of the glacial retreat has been known to impact species habitat occupancy (Gobbi et al. 2007). While some species of *Nebria* are still fully winged, many are brachypterous or flightless (Kavanaugh 1985), thus long‐distance dispersal is limited. During glacial maxima, alpine habitats shifted to lower elevations, and during glacial minima, the elevational range of riparian habitats lengthened and expanded up mountain slopes. Throughout glacial cycles, shifting habitats can allow species to more readily mix among adjacent mountain ranges, facilitating dispersal and possibly hybridization (Schoville et al. 2012). As glaciers retreat and riparian habitats expand upwards, microhabitat partitioning may act as an environmental filter on body proportions and body size as ecologically important traits under selection (Brehm et al. 2018; Mena et al. 2020). As species expand their geographic range, selection upon allometric scaling may play an increasingly important role in environmental filtering and habitat partitioning. This may explain why the riparian morphotype is so stable despite habitat variability in riparian zones. As species' ranges expand or move upwards, they may face novel selective pressures influencing movement rate and body proportions (Yarwood et al. 2021). Allometric scaling has been shown to change as body size increases with elevation (but see Egset et al. 2012 and Shelomi 2012). This is consistent with finer scale (spatially) studies of *Nebria* species assemblages across elevations and habitat types in the northern Cascades Range (Schat et al. 2024).

In contrast, the lack of absolute similarity among alpine species may be due to weaker environmental filtering on morphotype. Microhabitat features, rather than general habitat characteristics, are predictive of invertebrate habitat occupancy (Sinclair et al. 2001; Gobbi et al. 2021). Despite more limited evidence for alpine convergence in this study, in previous work we have shown that alpine specialists exhibit niche conservatism in morphological shape when they become isolated across mountain ranges (Schat et al. 2022). This most likely arises as a form of stabilizing selection, although ancestral constraint should be explored through more careful phylogenomic analysis.

## 5 Conclusions and Future Directions

Within the radiation of species of *Nebria*, morphological variation is best predicted by a combination of habitat use and ancestral constraint. There is evidence for convergence among species of *Nebria* with respect to alpine and riparian habitat specialization, but k‐means clusters suggest phylogenetic relatedness does explain an important part of the pattern in morphospace. It is not surprising that ancestral constraint predicts some of the morphological variation among closely related species. However, the importance of habitat use in predicting morphological variation, the morphological similarity of ecotypes, and the consistency of stabilizing selection upon these ecomorphological traits indicates that convergent processes significantly impact phenotypic variation. Further investigation of evolutionary history of additional ecomorphological traits, such as gross body proportions, may prove fruitful. Microhabitat use is an aspect of niche not closely examined in this study, and variation in gross body size proportions may be predictive of fine‐scale habitat use.

The methods used in this study employ comparative approaches, designed to test whether traits are predictably associated with their function while controlling for phylogenetic history. In future studies, optimality approaches, such as is discussed in Hansen (1997) and demonstrated in studies such as Bravo et al. (2014), may further illuminate the relationship between convergence and stabilizing selection in this system. In addition, more effort should be placed in examining convergence at the microhabitat scale. Microhabitats are subsets of habitats, which maintain more stable climatic conditions on short time scales (Scheffers et al. 2014). Most publicly available environmental data is only accurate to the nearest ~1km^2^ and is insufficient for describing microhabitat variation. Sampling habitat variability within these regions would provide insight into the potential habitat variation where species of *Nebria* are found. During periods of rapid climate change, these pockets of stability may become important aspects of environmental filtering. Ground beetles are useful indicators of significant habitat change (Messer 2009; Schirmel et al. 2015). Understanding how they respond to environmental changes is key to improving conservation outcomes over long time scales.

## Author Contributions

**Jillian K. Schat:** data curation (equal), formal analysis (lead), funding acquisition (lead), investigation (equal), methodology (equal), project administration (supporting), supervision (supporting), writing – original draft (lead). **Elizabeth Ehlert:** data curation (equal), investigation (supporting). **David H. Kavanaugh:** data curation (equal). **Roman Yu Dudko:** conceptualization (equal). **Sean D. Schoville:** conceptualization (equal), investigation (equal), methodology (equal), project administration (equal), resources (lead), supervision (lead), writing – review and editing (lead).

## Conflicts of Interest

The authors declare no conflicts of interest.

## Supporting information


Appendix S1.

## Data Availability

Morphometric data, specimen photographs, and R code used in this study are open access: https://datadryad.org/stash/share/jCP_ddXVyYhwkUlMXYzL6hVjA_y_n2a59HosWB‐W5Go.
