# Supplementary material for: Ecomorphological Convergence Following Niche Shifts in Montane Ground Beetles (Carabidae: Nebria)
Source: Ecol Evol. 2025 Feb 19;15(2):e70986. doi: 10.1002/ece3.70986 (PMC11839267; doi:10.1002/ece3.70986)
Supplement: Supplementary file 1 — Appendix S1. [file ECE3-15-e70986-s001.docx]

Appendix 1: Supplementary Material

Table 1 Sample sizes of species of *Nebria* analyzed in this study.

| Species | Sample Size |
| --- | --- |
| *Nebria acuta* Lindroth | 20 |
| *Nebria albimontis* Kavanaugh | 13 |
| *Nebria appalachia* Darlington | 17 |
| *Nebria arkansana* Casey | 20 |
| *Nebria balli* Kavanaugh | 19 |
| *Nebria bellorum* Kavanaugh | 10 |
| *Nebria beverlianna* Kavanaugh | 19 |
| *Nebria calva* Kavanaugh | 20 |
| *Nebria carri* Kavanaugh | 20 |
| *Nebria cascadensis* Kavanaugh | 20 |
| *Nebria castanipes* Kirby | 10 |
| *Nebria catenata* Casey | 20 |
| *Nebria charlottae* Lindroth | 20 |
| *Nebria chuskae* Kavanaugh | 10 |
| *Nebria coloradensis* Van Dyke | 12 |
| *Nebria crassicornis* Van Dyke | 9 |
| *Nebria danmanni* Kavanaugh | 18 |
| *Nebria darlingtoni* Kavanaugh | 19 |
| *Nebria desolata* Kavanaugh | 18 |
| *Nebria diversa* LeConte | 20 |
| *Nebria edwardsi* Kavanaugh | 20 |
| *Nebria eschscholtzii* Ménétriés | 20 |
| *Nebria fragariae* Kavanaugh | 20 |
| *Nebria fragilis* Casey | 20 |
| *Nebria frigida* Sahlberg | 19 |
| *Nebria gebleri* Dejean | 20 |
| *Nebria giulianii* Kavanaugh | 17 |
| *Nebria gouleti* Kavanaugh | 19 |
| *Nebria gregaria* Fischer von Waldeim | 16 |
| *Nebria gyllenhali* Schӧnherr | 20 |
| *Nebria haida* Kavanaugh | 20 |
| *Nebria hudsonica* LeConte | 20 |
| *Nebria ingens* Horn | 19 |
| *Nebria intermedia* Van Dyke | 10 |
| *Nebria jeffreyi* Kavanaugh | 16 |
| *Nebria kincaidi* Schwarz | 19 |
| *Nebria labontei* Kavanaugh | 15 |
| *Nebria lacustris* Casey | 10 |
| *Nebria lamarckensis* Kavanaugh | 19 |
| *Nebria lassenensis* Kavanaugh | 10 |
| *Nebria lindrothi* Kavanaugh | 10 |
| *Nebria lituyae* Kavanaugh | 20 |
| *Nebria louiseae* Kavanaugh | 20 |
| *Nebria lyelli* Van Dyke | 20 |
| *Nebria mannerheimii* Fischer von Waldheim | 20 |
| *Nebria meanyi* Van Dyke | 18 |
| *Nebria metallica* Fischer von Waldheim | 17 |
| *Nebria modoc* Kavanaugh | 13 |
| *Nebria navajo* Kavanaugh | 18 |
| *Nebria nivalis* Paykull | 9 |
| *Nebria obliqua* LeConte | 8 |
| *Nebria oowah* Kavanaugh | 20 |
| *Nebria ovipennis* LeConte | 20 |
| *Nebria pallipes* Say | 20 |
| *Nebria paradisi* Darlington | 19 |
| *Nebria pasquineli* Kavanaugh | 20 |
| *Nebria piperi* Van Dyke | 19 |
| *Nebria piute* Erwin & Ball | 19 |
| *Nebria purpurata* LeConte | 20 |
| *Nebria quileute* Kavanaugh | 19 |
| *Nebria rathvoni* LeConte | 20 |
| *Nebria riversi* Van Dyke | 5 |
| *Nebria sahlbergii* Fischer von Waldheim | 20 |
| *Nebria schwarzi* Van Dyke | 20 |
| *Nebria sevieri* Kavanaugh | 20 |
| *Nebria sierrablancae* Kavanaugh | 18 |
| *Nebria sierrae* Kavanaugh | 19 |
| *Nebria siskiyouensis* Kavanaugh | 20 |
| *Nebria sonorae* Kavanaugh | 13 |
| *Nebria spatulata* Van Dyke | 20 |
| *Nebria steensensis* Kavanaugh | 20 |
| *Nebria suturalis* LeConte | 20 |
| *Nebria sylvatica* Kavanaugh | 20 |
| *Nebria triad* Kavanaugh | 19 |
| *Nebria trifaria* LeConte | 20 |
| *Nebria vandykei* Bänninger | 20 |
| *Nebria wallowae* Kavanaugh | 19 |
| *Nebria wyeast* Kavanaugh | 20 |
| *Nebria zioni* Van Dyke | 8 |


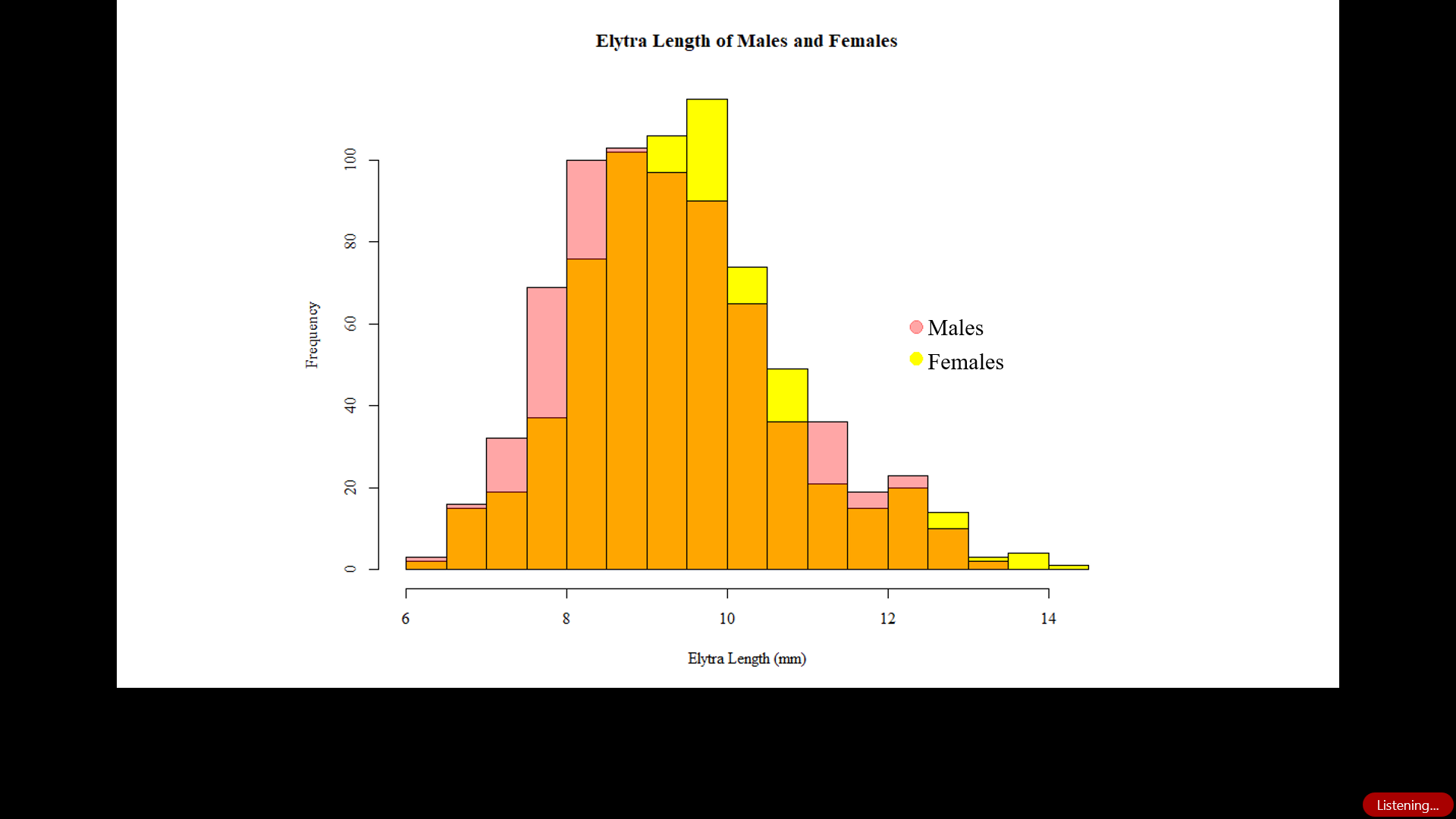


Figure 1 Elytral length of males and females across species of *Nebria*. Males are indicated by light red, and females are indicated by yellow. While elytral length is statistically discernable between males and females, there is high overlap in the distribution of these values across sexes.


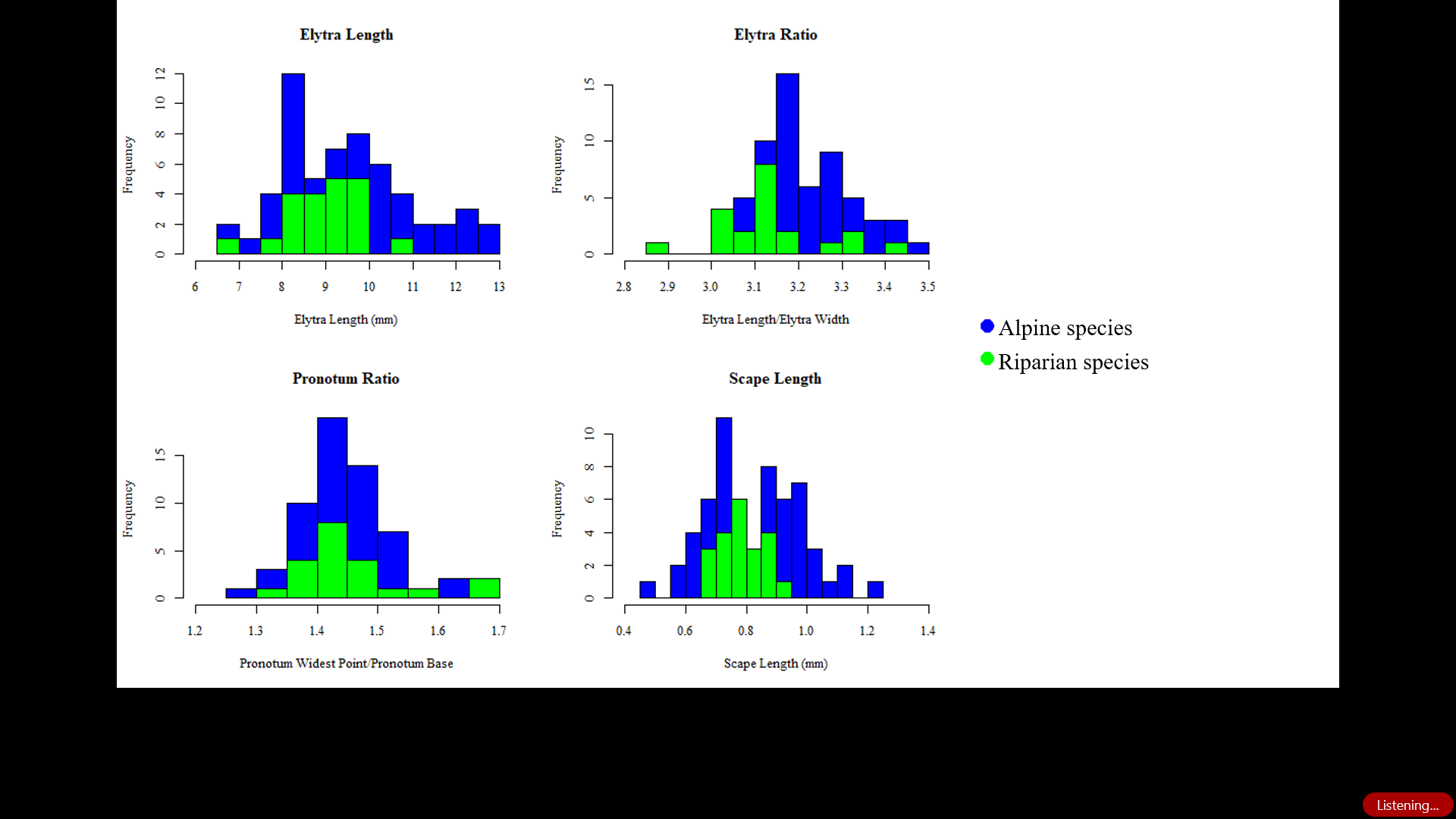


Figure 2 Linearly measured morphological traits of species of *Nebria*. Colors indicate whether species were collected in alpine or riparian habitats based on a threshold mean ground temperature of 6˚C.


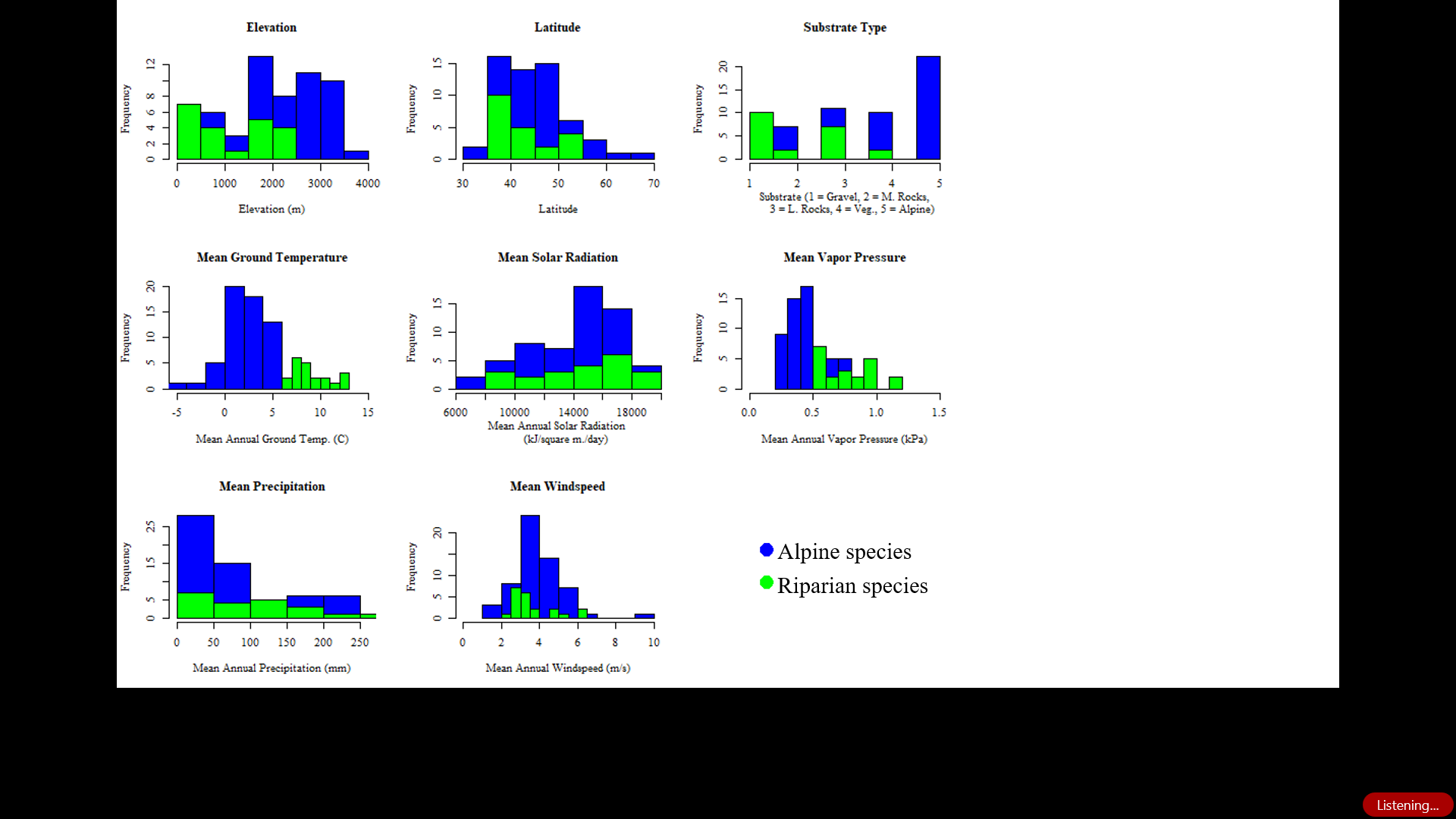


Figure 3 Habitat characteristics where species *Nebria*. were collected using the Worldclim database (Fick & Hijmans, 2017). Colors indicate whether species were collected in alpine or riparian habitats based on a threshold mean ground temperature of 6˚C.


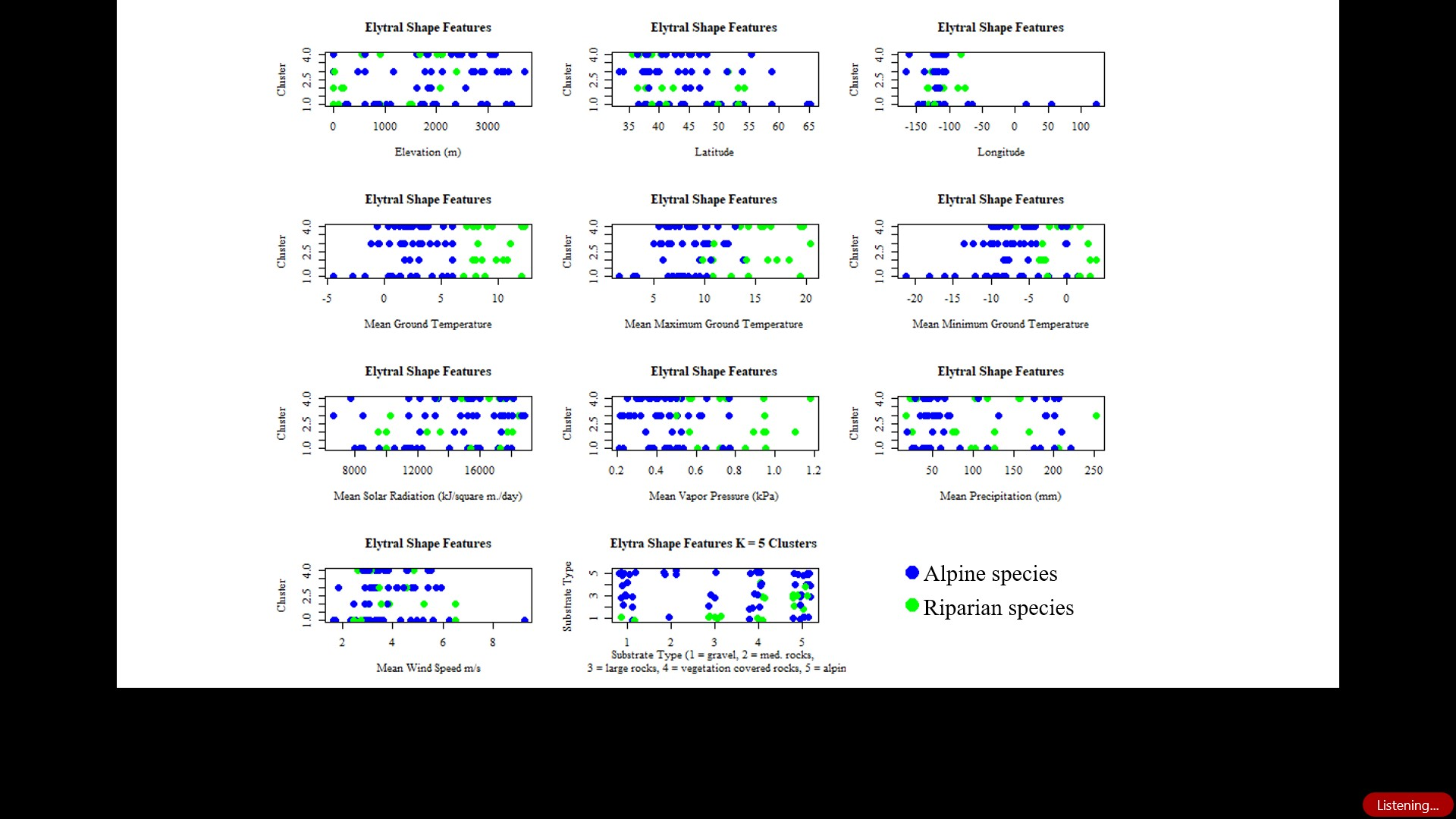


Figure 4 Elytral shape features formed k = 4 clusters in morphospace. These clusters do not appear to correspond to variation in habitat use. Colors indicate whether species were collected in alpine or riparian habitats based on a threshold mean ground temperature of 6˚C. For comparison with substrate type, elytral shape features were coerced to five clusters, corresponding to the categories of substrate type used in this study.


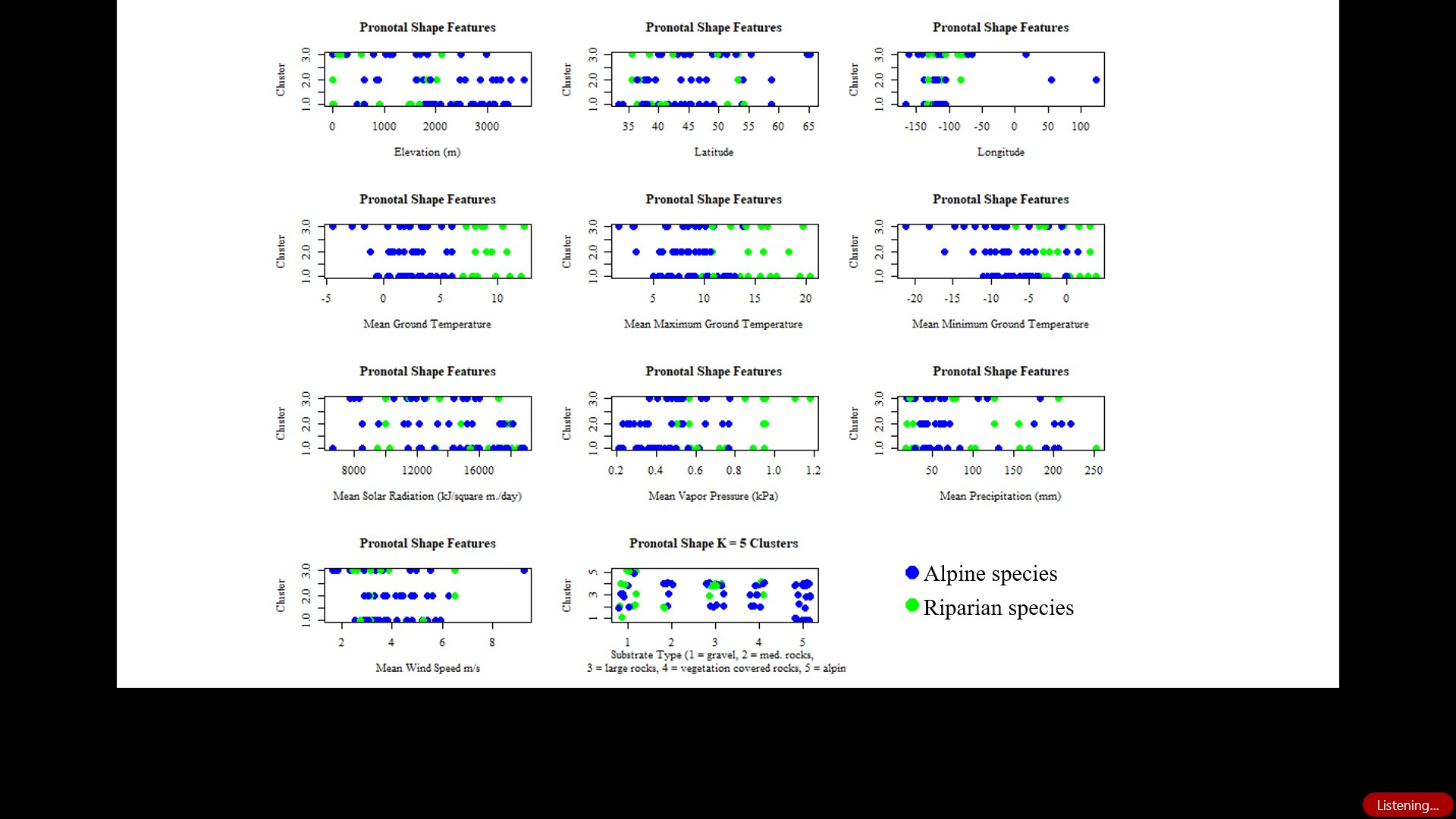


Figure 5 Pronotal shape features formed k = 3 clusters in morphospace. These clusters do not appear to correspond to variation in habitat use. Colors indicate whether species were collected in alpine or riparian habitats based on a threshold mean ground temperature of 6˚C. For comparison with substrate type, pronotal shape features were coerced to five clusters, corresponding to the categories of substrate type used in this study.


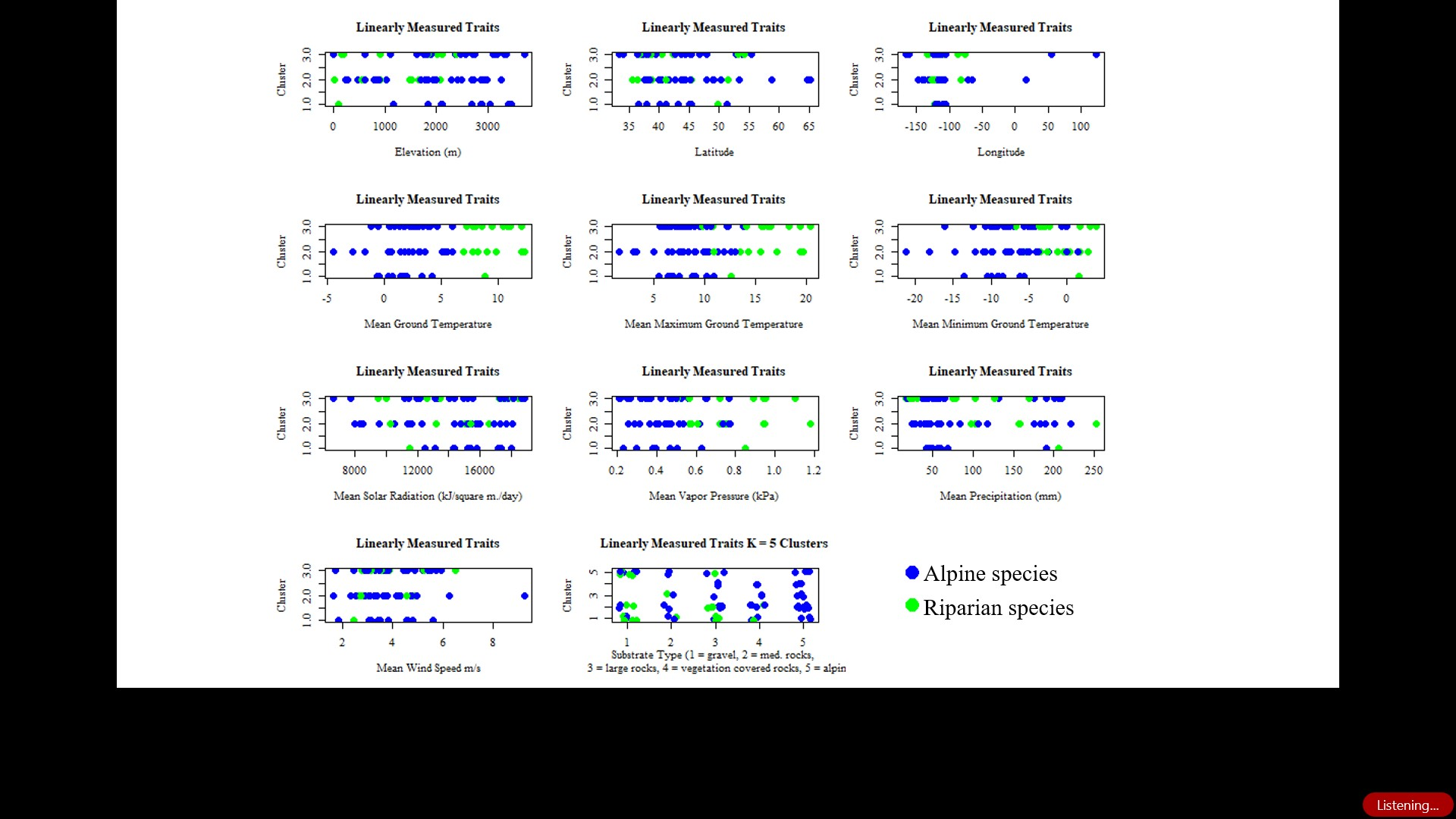


Figure 6 Linearly measured traits formed k = 3 clusters in morphospace. These clusters do not appear to correspond to variation in habitat use. Colors indicate whether species were collected in alpine or riparian habitats based on a threshold mean ground temperature of 6˚C. For comparison with substrate type, linearly measured traits were coerced to five clusters, corresponding to the categories of substrate type used in this study.
